# Impact of Age on tDCS Effects on Pain Threshold and Working Memory: Results of a Proof of Concept Cross-Over Randomized Controlled Study

**DOI:** 10.3389/fnagi.2020.00189

**Published:** 2020-06-30

**Authors:** Júlia Schirmer Saldanha, Maxciel Zortea, Cibely Bavaresco Deliberali, Michael A. Nitsche, Min-Fang Kuo, Iraci Lucena da Silva Torres, Felipe Fregni, Wolnei Caumo

**Affiliations:** ^1^Graduate Program in Medical Sciences, School of Medicine, Universidade Federal do Rio Grande do Sul (UFRGS), Porto Alegre, Brazil; ^2^Laboratory of Pain & Neuromodulation, Clinical Research Center, Hospital de Clínicas de Porto Alegre (HCPA), Porto Alegre, Brazil; ^3^Leibniz Research Centre for Working Environment and Human Factors, Dortmund, Germany; ^4^Department of Neurology, University Medical Hospital Bergmannsheil, Bochum, Germany; ^5^Department of Pharmacology, Instituto de Ciências Básicas da Saúde, Universidade Federal do Rio Grande do Sul (UFRGS), Porto Alegre, Brazil; ^6^Pharmacology of Pain and Neuromodulation: Pre-clinical Investigations Research Group, Universidade Federal do Rio Grande Do Sul (UFRGS), Porto Alegre, Brazil; ^7^Laboratory of Neuromodulation and Center for Clinical Research Learning, Physics and Rehabilitation Department, Spaulding Rehabilitation Hospital, Boston, MA, United States; ^8^Pain and Palliative Care Service, Hospital de Clínicas de Porto Alegre (HCPA), Porto Alegre, Brazil; ^9^Department of Surgery, School of Medicine, Universidade Federal do Rio Grande do Sul (UFRGS), Porto Alegre, Brazil

**Keywords:** transcranial direct current stimulation (tDCS), heat pain threshold, working memory, age, adolescents, elderly, quantitative sensory testing

## Abstract

**Background**: Age is an important factor that impacts the variability of tDCS effects.

**Objective/Hypothesis**: To compare effects of anodal (a)-tDCS over the left dorsolateral prefrontal cortex (DLPFC), and primary motor cortex (M1) in adolescents, adults, and elderly on heat pain threshold (HPT; primary outcome) and the working memory (WM; secondary outcome). We hypothesized that the effect of tDCS on HPT and WM performance would be the largest in adolescents because their pre-frontal cortex is more prone to neuroplasticity.

**Methods**: We included 30 healthy women within the age ranges of 15–16 (adolescents, *n* = 10), 30–40 (adults, *n* = 10), and 60–70 (elderly, *n* = 10) years. In this crossover single-blinded study, participants received three interventions applied over the DLPF and M1. The active stimulation intensity was two mA for 30 min. From 20 min of stimulation onset, the tDCS session was coupled with an online n-back task. The a-tDCS and sham were applied in a random sequence, with a washout time of a minimum 7 days between each trial. HPT was evaluated before and after stimulation. The WM performance with an n-back task was assessed after the tDCS session.

**Results**: A Generalized Estimating Equation (GEE) model revealed a significant effect of the a-tDCS over the left DLPFC to reduce the HPT in adolescents compared with sham. It increased the pain perception significantly [a large effect size (ES) of 1.09)]. In the adults, a-tDCS over M1 enhanced the HPT significantly (a large ES of 1.25) compared to sham. No significant effect for HPT was found in the elderly. Response time for hits was reduced for a-tDCS over the DLPFC in adolescents, as compared to the other two age groups.

**Conclusions**: These findings suggest that a-tDCS modulates pain perception and WM differentially according to age and target area of stimulation. In adolescents, anodal stimulation over the DLPFC increased the pain perception, while in adults, the stimulation over the M1 increased the pain threshold. Thus, they elucidate the impact of tDCS for different age groups and can help to define what is the appropriate intervention according to age in further clinical trials.

**Clinical Trial Registration:** www.ClinicalTrials.gov, Identifier: NCT04328545.

## Introduction

Transcranial direct current stimulation (tDCS) modulates cortical excitability with a low-intensity continuous electric current applied *via* two or more electrodes placed on the scalp (Nitsche and Paulus, 2000; Nitsche et al., 2008). Anodal (a)-tDCS induces neuronal membrane depolarization at critical neural compartments and enhances cortical excitability while cathodal tDCS decreases the excitability of respective target areas. The tDCS effects involve synaptic plasticity mechanisms of glutamatergic synapses that resemble the long-term potentiating (LTP) or the long-term depression (LTD; Nitsche et al., 2008). Anodal tDCS over the left dorsolateral prefrontal cortex (DLPFC) and primary motor cortex (M1) has been shown to decrease pain levels in chronic pain patients and increase the pain threshold in adults’ healthy subjects (Vaseghi et al., 2014; Zortea et al., 2019). The a-tDCS effect over M1 is attributed to modulating the sensory discrimination of pain, such as threshold, quality, location, and intensity (Lorenz et al., 2003; Boggio et al., 2008). Prefrontal stimulation is believed to modulate affective-emotional and cognitive aspects of pain (Boggio et al., 2008). This effect’s biological plausibility is supported by prefrontal connections to the anterior cingulate cortex (ACC), insula, and amygdala. Besides, the a-tDCS over the DLPFC can improve several cognitive domains such as perception, attention, working memory, learning, and decision-making (Teixeira-Santos et al., 2015). This area’s stimulation modulates a core circuit of working memory on the frontoparietal cortical regions (Fregni et al., 2005). However, these general effects of the tDCS efficacy are not entirely homogeneous (Fertonani and Miniussi, 2017; Mahdavi and Towhidkhah, 2018). They depend on anatomical factors (e.g., gray matter density, cytoarchitecture, baseline activity/excitability state), and, neuroplasticity intrinsic factors, such as genetics, sex, time of day, cognitive involvement, and age (Ridding and Ziemann, 2010). The chronological age has been suggested as the main factor for the inter-individual variability of neuromodulatory effects (Ridding and Ziemann, 2010; Freitas et al., 2013).

Even though the tDCS evidence in the therapeutic field is growing, as well as age is known as a determinant of neuroplasticity, the majority of mechanistic tDCS studies have been conducted in young adults. Thus, a gap in the literature remains related to age as a mediator of the tDCS effect. Notably, there is a scarcity of studies systematically designed to evaluate the tDCS effect according to the stimulation area for different age groups (e.g., adolescents, adults, or elderly). This rationale is supported in the acknowledgment that during adolescence, the prefrontal cortex is undergoing a maturation process which comprises synaptic pruning and myelinization (Sowell et al., 2004). In contrast, in the elderly, the senescence processes include a reduction of neuronal size and decrease of gray matter (Resnick et al., 2003). Hence, we need to better comprehend age as a factor that could mediate the tDCS effects according to the stimulation area.

Thus to elucidate the impact of age as a mediator on the tDCS effects, we conducted this randomized explanatory, sham-controlled, single-blind crossover study to test our hypothesis under an experimental paradigm where we control the etiological components of pain (e.g., nature, localization, intensity, frequency, and duration of the trigger necessary to evoke pain). We aimed to compare the effects of anodal tDCS applied over the left DLPFC and M1 in different age groups (adolescents, adults, and elderly) on the heat pain threshold (HPT; primary outcome). Also, we evaluated their effects on the heat threshold, heat pain tolerance, and moderate heat pain and working memory (WM) as secondary outcomes. Due to the higher propensity for neuroplasticity of the prefrontal cortex in adolescents, and the plasticity-dependency of tDCS effects, we hypothesized that a-tDCS in adolescents would elicit the largest variation when compared to adults and elderly and that the differences of tDCS effects would be more prominent for DLPFC stimulation.

## Materials and Methods

### Study Design

A randomized, single-blinded, and sham-controlled cross-over study was conducted. The Institutional Review Board approved the study of the Hospital de Clínicas of Porto Alegre, Brazil (IRB HCPA/Approval number: 170188). De-identified data concerning intervention and outcomes will be made available upon reasonable request to WC (wcaumo@hcpa.edu.br). All participants provided written informed consent following the Declaration of Helsinki. For the participants under the legal age, the written consent was signed by parents or legal guardian and by the participants themselves. The recruitment period was from September 2017 to December 2019.

**Figure 1:**
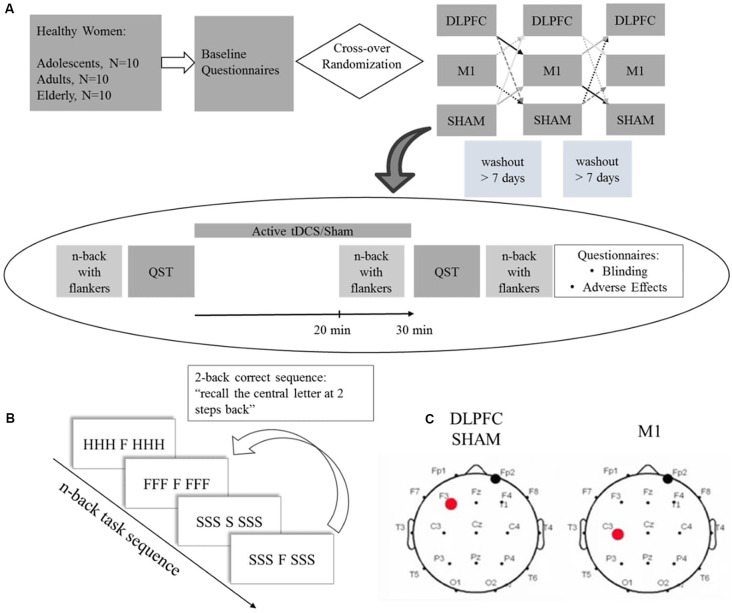
**(A)** Experimental protocol timeline. Baseline questionnaires included the Beck Depression Inventory II (BDI-11), Pittsburgh Sleep Quality Index (PSQI), State-Trait Anxiety Inventory (STAI), and a standardized demographic questionnaire. **(B)** Scheme depicting a 2-back task stimuli sequence with congruent and incongruent flankers. **(C)** tDCS montage for dorsolateral prefrontal cortex (DLPFC), Sham and primary motor cortex (M1), the red dot represents the anode electrode, and the black dot the cathode electrode. QST = Quantitative Sensory Test.

Study volunteers were recruited from the community and enrolled after inclusion and exclusion criteria screening. After the enrollment, participants filled a standardized assessment to assess sociodemographic characteristics, anxiety, sleep quality, and depressive symptoms. Participants were randomized for a cross-over order of intervention for three sessions: a-tDCS over DLPFC, a-tDCS over M1, and sham. The baseline n-back performance was assessed in the first session. For each cross-over trial, participants followed the study protocol depicted in Figure 1: Quantitative Sensory Test (QST) test was performed at first, followed by 30 min active tDCS or sham coupled with the n-back task for the last 10 min. The n-back task was assessed after the stimulation QST and followed by blinding and adverse effects questionnaires. The timeline of the study is presented in Figure 1.

### Participants and Sample Size

The subjects were recruited from the general population by advertisement postings in public places in Porto Alegre, Brazil. We recruited 30 voluntary female participants of three age groups: adolescents between 15 and 16 years old, adults between 30 and 40 years old, and elderly between 60 and 70 years old. Inclusion criteria: right-handedness, education level that ranged from incomplete middle school to incomplete undergraduate course (5–13 years of study), normal or corrected-to-normal visual acuity, post menarche for the adolescent and adult participants, and post-menopause (more than 12 months of amenorrhea according to ACOG (American College of Obstetricians and Gynecologists) for elderly participants. Exclusion criteria: use of neuropsychiatric medications; clinically significant or unstable medical, neuropsychiatric, or chronic pain condition; the history of substance abuse, smoking, stroke, epilepsy, brain surgery, implants, or brain tumor, pregnancy. We included only female participants since sex influences pain sensitivity (Bartley and Fillingim, 2013) and also modulated the tDCS effect on cortical excitability for cathodal, and anodal stimulation in both stimulation areas, M1 and DLPFC (Kuo et al., 2006; Lee et al., 2018).

The estimated prior sample size for the HPT was based on previous studies (Fregni et al., 2005; Graff-Guerrero et al., 2005; Mylius et al., 2012). This initial sample size was estimated by a difference of 4.5% between intervention groups applied over either stimulation sites, DLPFC, and M1. For this initial estimation, we used the PASS 2020 software^1^. Based on a generalized estimating equations (GEE) model in a repeated measures design, by a power of 80% and critical alpha set at 0.05, we required eight subjects per group. However, we planned interim analysis after enrollment of the first ten subjects in each group. Following the *O’Brien and Fleming criteria* (O’Brien and Fleming, 1979), we pre-specified that the trial would be stopped if there was a significant difference in the primary outcome (HPT) between intervention groups according to age categories, irrespective of stimulation site (*P* < 0.05). Considering that the sample size calculation was just an estimation, the calculation of power for this study by two independent researchers had been planned as part of the study design after enrollment of the first 30 subjects because this would indicate the effectiveness of the intervention. Based on the GEE model, the power of analysis to detect a difference in the HPT with a large effect size (Cramer’s V equal to 0.52), considering the interaction age group × stimulation condition, was 83% for a critical alpha of 0.05 (G*Power 3.0.10, Franz Faul, Universität Kiel, Germany).

### Intervention

Participants underwent two a-tDCS sessions over the respective areas of interest defined according to the EEG 10-20 system (Figure 1), and sham stimulation. For anodal DLPFC stimulation and sham, the electrode was placed over the left DLPFC (F3) and the cathode over the contralateral supraorbital area (FP2). For M1 stimulation, the anode was placed over the left M1 (C3), and the cathode over the contralateral supraorbital area. Stimulation was applied by the StarStim device (Neuroelectrics Barcelona SL, Spain) with 25 cm^2^ round sponges soaked with 0.9% saline solution. In the active conditions, a current of 2 mA was applied for 30 min with a ramp up and down of 30 s. For the sham protocol, no current was applied except for the 30 s ramp up and down.

### Randomization

The randomization order was generated using randomization.com. Ratio allocation was 1.1.1 for each tDCS montage and sham. Opaque envelopes containing session allocation were sealed and numbered sequentially. The envelopes were opened after gaining subjects’ informed and signed consent. All subjects were allocated to receive three sessions of tDCS (DLPFC a-tDCS, M1 a-tDCS, and sham). The minimum washout period was 7 days.

### Blinding

The device was programmed to deliver real (active) or sham stimulation before each application according to the participant’s code. Therefore, participants were blinded to the allocated interventions and were informed of the order of type of application was random. Researchers responsible for administering the intervention or conducting outcome assessments were not systematically blinded to the intervention. To assess if the blinding had been effective, we asked all participants to guess whether they had received a-tDCS or sham at the end of each one of the crossover trials.

### Instruments and Assessments

The participants completed the following questionnaires at baseline: Beck Depression Inventory-II (Gomes-Oliveira et al., 2012), Pittsburgh Sleep Quality Index (PSQI; Bertolazi et al., 2011) and State-Trait Anxiety Inventory (STAI; Kaipper et al., 2010). All these tests are validated for the Brazilian population. Demographic data were assessed using a standardized questionnaire.

### Outcomes

The primary outcome parameters were HPT. Secondary outcomes were the heat sensitivity threshold (HST), heat pain tolerance (HPTol), and moderate heat pain (HMP) and WM evaluated by D-prime.

#### Quantitative Sensory Testing

The QST uses a Peltier thermode (30 × 30 mm) to assess HPT according to the method of limits (Schestatsky et al., 2011). For the assessment of HTP, the thermode was placed on the ventral right forearm. The baseline temperature was 30°C. It was increased at 1°C/s intervals up to a maximum of 52°C. HPT is the temperature at which volunteers first indicate pain. HST is the temperature at which the volunteers first report a heat sensation. The HPTol is the temperature that induces the maximum tolerated pain. The HMP is the temperature equivalent to the value six on a Visual Analogue Pain Scale (scores reaching from 0 to 10). A computerized Brazilian version of the QST test was used (Heat Pain Stimulator 1.1.10, Brazil developed by the Pain and Neuromodulation Laboratory). The quantitative Sensory Test (QST) was evaluated before and after the tDCS or sham interventions for each one crossover trial.

#### Working Memory

We used an n-back task coupled with flankers to assess working memory performance (adapted from Scharinger et al., 2015). It was evaluated by D-prime, which is a sensitivity parameter for accuracy. The participants performed a computerized n-back task composed of three blocks of different task workloads: zero-back, one-back, and two-back. The n-back test consists of a series of letters presented consecutively, and the participants should respond if the present letter is equal to the letter presented “n” letters previously (Figure 1B). The “n” represents the number indicating how many previous trials the participant should remember the presented letters: one-back corresponds to recall of the letter presented one trial before, two-back to the letter presented two trials before. If the stimulus is identical to “n” previous trials, the participant must indicate this by pressing the mouse button as fast as possible. For the zero-back, a letter is presented at the beginning of the task, and the participants should indicate when the letter of a specific trial is equal to that letter. We applied the task with flankers. Here the cue letter is displayed in the center, combined with three other letters on each side. The side letters had two conditions: congruent and incongruent. The congruent condition occurs when the same letter appears on the right and left sides of the target letter, for example, CCC C CCC. In the incongruent condition, the letters are different, for example, SSSH SSS. Participants were instructed to focus only on the letter presented centrally (that is, ignore the flanker). The test sequences within the blocks were pseudorandomized. The task was created using E-Prime software version 2.0 SP1 (Psychology Software Tools, Sharpsburg, PA, USA). The baseline n-back task was assessed on the first crossover session. Participants performed an n-back task during stimulation for the last 10 min and were evaluated for WM performance with an n-back task after tDCS.

### Assessment of Blinding and Adverse Effects

A structured questionnaire was used to assess the most frequent adverse effects after each one crossover trial, as well as classify their severity as mild, moderate, or severe (Supplementary Material). Also, there was an open question to describe any adverse effects, not presented in the list.

The subjects’ blinding to intervention (a-tDCS or sham) was evaluated by a questionnaire where participants should indicate if a sham or active stimulation was performed. Participants answered this standardized questionnaire to evaluate their blinding after each one intervention session for all three crossover trials.

### Statistical Analysis

We conducted descriptive analyses with mean and standard deviation (SD) for continuous variables. To compare baseline characteristics between groups, we applied one-way ANOVA. The incidence and severity of adverse effects as well as blinding to intervention (a-tDCS and sham condition) were compared by Fisher’s exact test. To assess the changes on the HPT from before intervention (T0) to after (T1) tDCS the percentage of variation [(T1 − T0)/T0]*100 was used. A generalized estimating equations (GEE) model was used to compare the effect of interventions considering four factors: [group (adolescents, adults, elderly), stimulation condition (a- TDCS over DLPFC, a-tDCS over M1, or sham), the interaction group*stimulation condition and order of intervention]. A GEE model was used to evaluate the effect of the intervention on working memory performance assessed by D-prime (D prime = hit rate minus false alarm rate) and RTH. The model considered four factors: (age group, stimulation condition, interaction group*stimulation condition, and order of intervention). The GEE models for the D-prime and RTH were adjusted by the n-back task at baseline performance and years of study. All analyses were adjusted for multiple comparisons by Bonferroni test and Dunn’s method for Kruskal-Wallis tests when appropriate. The critical *p*-value was set to 5% for all statistical tests. The data were analyzed using SSPS version 22.0 (SPSS, Chicago, IL, USA).

## Results

### Sample Characteristics

**Figure 2:**
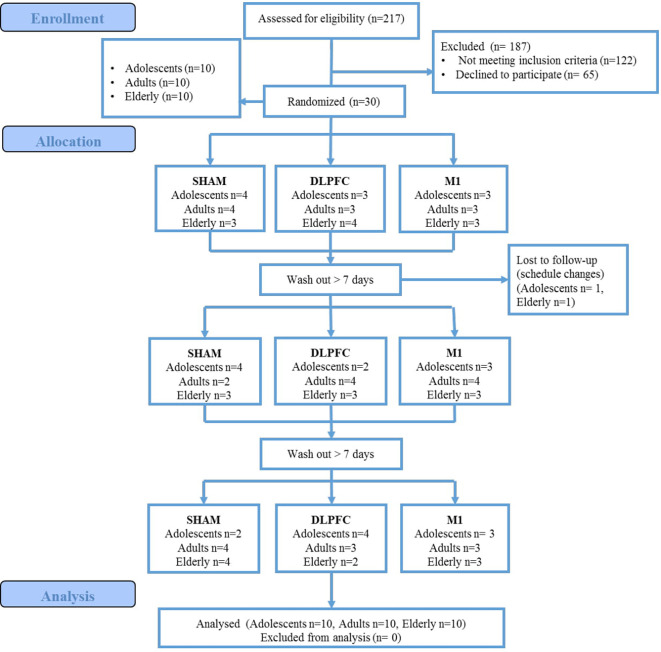
CONSORT participants flow.

A total of 217 women were assessed for eligibility, 122 did not meet the inclusion criteria (history of chronic pain, or university degree), and 65 declined to participate. These data are presented in the participants’ flow (Figure 2). Thirty female participants were enrolled, 10 for each age group (adolescents, adults, and elderly). The blinding of the participants related to the intervention was successful, and there was no significant difference in the guessing for active stimulation or sham (for details refer to Supplementary Material). We did not find statistically significant differences in the incidence or intensity of adverse effects between stimulation protocols within age groups (for details refer to Supplementary Material). Between age groups, the sleepiness for a-tDCS over the DLPFC and sham-tDCS showed that the elderly presented a higher incidence. In the sham condition, sleepiness was reported by the elderly (50%) by adults (10%), but not by adolescents (*P* = 0.026). For a-tDCS on the DLPFC, the sleepiness was reported to either elderly (55%), and adults (10%) but not in adolescents (*P* = 0.014). There was no difference in the sleepiness among groups for the tDCS applied to the M1. Regarding the severity of adverse effects, for LDPFC, there was a significant difference between age groups in the itching severity (*P* = 0.01). Itching was reported by adolescents (80%), who in totality classified it as moderate. Adults also reported itching (87.5%), and 62.5% of them classified it as moderate to severe. In the elderly, 44% reported itching, and all of them classified it as mild. In our study, 45% of all participants, including adolescents and adults, were using some form of hormonal contraceptive.

The demographic and psychological data of the three age groups are presented in Table 1. Differences between groups were only found for years of study (*P* = 0.001).

**Table 1:** Demographic and psychological characteristics of the age groups.

	Adolescents (*n* = 10)	Young adults (*n* = 10)	Elderly (*n* = 10)	*P*-value
Age (years)	15.6 (0.5)	33.9 (3.3)	63.8 (2.6)	-
Years of study	9.2 (1.1)	12.9 (1.7)	10.2 (2.6)	0.001
Beck Depression Inventory II	13.6 (12.1)	10.1 (9.4)	10.8 (6.7)	0.697
State-Trait Anxiety Inventory State	23.6 (4.5)	22.5 (3.4)	20.5 (4.7)	0.091
State-Trait Anxiety Inventory Trait	21.7 (4.7)	19.4 (4.3)	19.2 (4.2)	0.345
Pittsburgh Sleep Quality Index	5.7 (2.6)	5.4 (3.0)	5.5 (3.2)	0.974

*Mean differences between groups were analyzed by one ANOVA. The data are presented as mean and standard deviation (SD; *n* = 30)*.

### Effect of tDCS Applied Over M1 and DLPFC on the Primary Outcome

A GEE model showed no order effect across the study for the HPT (*P* = 0.35). The GEE model revealed a statistically significant effect for age group*stimulation condition interaction (Wald *χ*^2^ = 16.613; *df* = 4; *P* < 0.01). In adolescents, the a-tDCS over the DLPFC compared to sham increased the pain perception. This finding indicates on the HPT was in the opposite direction than the effect in adults and elderly with a-tDCS applied over the same stimulation site. The between-age group test, according to the stimulation condition revealed that the a-tDCS applied over the DLPFC in the HPT variation in adolescents was significantly higher than the elderly. The effect size (ES) within adolescents’ groups of the a-tDCS over DLPFC compared to sham was 1.09 as assessed by Cohen’s D. The ES between adolescents compared to adults and the elderly with a-tDCS on the DLPFC was 0.97 and 1.64, respectively.

In adults, the a-tDCS over M1 compared to sham increased the HTP with ES equal to 1.25. The active stimulation on M1 in adults increased the pain threshold of 48.86% compared to the DLPFC. However, this difference was not statistically significant (Table 2). Regarding the tDCS effect on HPT with stimulation on the M1 did not differ statistically between age groups.

**Table 2:** Heat pain thresholds by quantitative Sensory Test (QST), and percentage of change from before (B) to after (A), according to age groups and stimulation site.

Primary outcome [-8pt]	Adolescents^(1)^				Adults^(2)^				Elderly^(3)^			
	Mean (SD)				Mean (SD)				Mean (SD)			
	Before (B)	After (A)	Mean change (B to A) (%)^$^		Before (B)	After (A)	Mean change (B to A) (%)**^$^**		Before (B)	After (A)	Mean change (B to A) (%)^$^	
Heat Pain Threshold (HPT)^†^				ES				ES				ES
Sham	40.9 (3.1)	41.5 (3.7)	1.51% (5.0)	-	42.8 (2.4)	41.5 (2.4)	−2.68% (5.4)	-	41.7 (3.1)	41.9 (2.5)	0.70% (7.3)	-
DLPFC^2, 3^	42.3 (3.1)	40.8 (3.2)	−3.38% (3.8)	1.09	41.6 (1.5)	42.2 (1.4)	1.62% (6.1)	0.74	43.5 (2.6)	44.4 (2.6)	2.24% (3.0)	0.27
M1^2^	40.3 (3.0)	41.5 (3.2)	3.09% (5.8)	0.29	42.2 (2.0)	43.8 (2.4)	3.91% (5.1)	1.25	41.4 (3.5)	42.2 (2.9)	1.66% (7.1)	0.12
Stimulation condition*age group: Wald *χ*^2^ = 16.613; df = 4; P = 0.002.												
*Order of stimulation: Wald χ = 2.10; df = 2; P = 0.350*.												
*Stimulation condition: Wald χ = 5.25; df = 2; P = 0.073*.												
*Age group: Wald χ = 0.87; df = 2; P = 0.648*.												

*^$^Mean difference in the on mean change before (B) to after (A) presented as mean change (%) according to age group (adolescents, adult elderly) and intervention groups (tDCS vs. sham). ^†^GEE model; Celsius (°C). Notes: Post-hoc differences between groups are indicated *via* superscript numbers, which correspond to the respective groups which are labeled as 1–3. Mean difference groups. Effect size (ES; Percentage of mean change on sham group minus a percentage of mean on a-tDCS over DLPFC or M1)/Standard deviation on sham. The effect size was defined as small if lower than 0.20; moderate if between 0.50–0.60; and large if larger than 0.80. Data are presented as mean and standard deviation (SD; *n* = 30)*.

**Figure 3:**
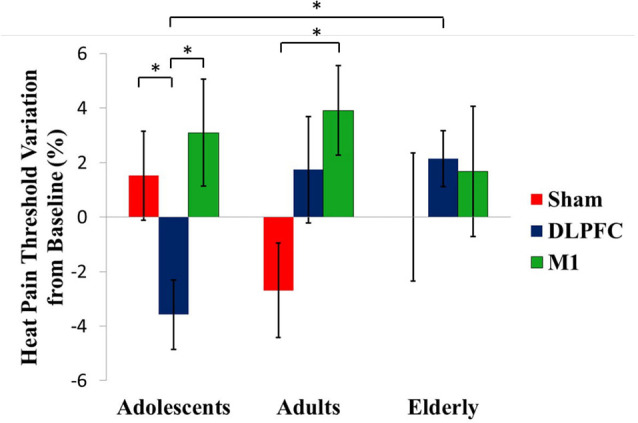
tDCS-driven heat pain threshold (HPT) alterations in the different age groups. Asterisks indicate statistical significance (*P* < 0.05). Error bars represent standard error of means (SEM).

Figure 3 presents the percentage of variation in the HPT according to the age group and interventions group with the comparisons within and between by a GEE model.

### Effect of tDCS Applied Over M1 and DLPFC on Secondary Outcomes

#### Effects on Pain Measures

The results of the GEE model showed no effect of stimulation in any of the age groups for these pain measures: heat threshold, heat pain tolerance, and moderate heat pain (Table 3). The a-tDCS over the left DLPFC increased the pain perception measured by the heat moderate pain and HPTol. From the baseline, the temperature in Celsius Degrees to produce moderate heat pain and the HPTol reduced by −1.44%, and by −2.15%, respectively. Whereas in the other groups, the a-tDCS decreased the pain perception, that is, it needed higher temperatures to produce the same pain perception. In adults, to produce moderate heat pain and the HPTo, the temperature increased by 0.44%, and by 1.41%, respectively. In the elderly, the temperature to produce moderate heat pain and the HPTol increased by 2.87% and by 0.38%, respectively.

**Table 3:** QST-derived pain measures and percentage of change from before (B) to after (A), according to age groups and stimulation site. Data are presented as mean and standard deviation (SD; *n* = 30).

	Adolescents			Adults			Elderly		
	Mean (SD)			Mean (SD)			Mean (SD)		
	Before (B) treatment	After (A) treatment	Mean change (B to A) (%)^$^	Before (B) treatment	After (A) treatment	Mean change (B to A) (%)^$^	Before (B) treatment	After (A) treatment	Mean change (B to A) (%)^$^
**Heat Threshold^†^**									
Sham	35.7 (2.5)	37.9 (2.5)	6.59% (7.2)	36.3 (3.3)	37.7 (2.0)	4.13% (4.4)	38.8 (3.9)	39.0 (2.8)	1.04% (6.9)
DLPFC	37.3 (3.8)	38.2 (4.2)	2.54% (6.8)	36.4 (2.5)	37.6 (1.7)	3.41% (5.0)	38.9 (3.5)	39.8 (2.6)	2.59% (3.5)
M1	36.5 (2.5)	38.0 (2.9)	4.34% (6.1)	36.5 (2.6)	39.0 (2.5)	7.18% (4.9)	38.0 (3.1)	39.0 (3.1)	2.81% (7.9)
Stimulation condition*age group: Wald χ^2^ = 3.304; df = 4; P = 0.508.									
*Order of stimulation: Wald *χ*^2^ = 5.800; df = 2; P = 0.055*.									
*Stimulation condition: Wald *χ*^2^ = 3.038; df = 2; P = 0.219*.									
*Age group: Wald *χ*^2^ = 4.741; df = 2; P = 0.093*.									
**Heat Pain Tolerance^†^**									
Sham	44.3 (3.8)	43.9 (4.8)	−0.78% (7.8)	45.5 (1.7)	45.9 (2.5)	1.00% (4.5)	44.8 (2.6)	44.3 (2.5)	−0.80% (8.1)
DLPFC	45.6 (3.2)	44.6 (2.8)	−2.15% (2.7)	45.9 (0.8)	46.1 (1.4)	0.44% (3.4)	47.0 (1.9)	47.1 (2.8)	0.38% (3.9)
M1	44.8 (2.8)	45.9 (3.0)	2.40% (5.7)	46.8 (2.4)	47.5 (1.8)	1.70% (5.7)	43.9 (3.6)	44.5 (2.3)	1.77% (6.6)
Stimulation condition*age group: Wald χ^2^ = 2.203; df = 4; P = 0.698.									
*Order of stimulation: Wald *χ*^2^ = 1.756; df = 2; P = 0.416*.									
*Stimulation condition: Wald *χ*^2^ = 3.881; df = 2; P = 0.144*									
*Age group: Wald *χ*^2^ = 0.957; df = 2; P = 0.620*.									
**Moderate Heat Pain^†^**									
Sham	40.9 (2.8)	41.2 (4.0)	0.53% (4.8)	42.2 (2.8)	42.7 (1.7)	1.32% (4.3)	41.7 (3.4)	41.6 (1.8)	0.17% (6.5)
DLPFC	42.0 (3.9)	41.3 (2.9)	−1.44% (4.9)	41.8 (1.6)	42.4 (2.2)	1.41% (6.4)	43.4 (2.5)	44.7 (2.4)	2.87% (2.9)
M1	41.4 (2.9)	41.5 (3.8)	0.15% (6.7)	43.1 (2.9)	43.8 (2.6)	1.85% (6.0)	42.0(2.6)	42.7(2.2)	1.90% (5.9)
Stimulation condition*age group: Wald *χ*^2^ = 1.891; df = 4; P = 0.756.									
*Order of stimulation: Wald *χ*^2^ = 9.991; df = 2; P = 0.007*.									
*Stimulation condition: Wald *χ*^2^ = 0.054; df = 2; P = 0.973*.									
*Age group: Wald *χ*^2^ = 2.867; df = 2; P = 0.238*.									

*^$^Mean difference in the on mean change before (B) to after (A) presented as mean change (%) according to age group (adolescents, adult elderly) and intervention groups (tDCS vs. sham). ^†^GEE model; Celsius (°C)*.

#### Effects on Working Memory

##### D Prime—Univariate Analysis

The Kruskal–Wallis test was used to compare D prime for baseline n-back task performance evaluated at cross-over day 1. A significant difference between age groups was found in the two-back task with congruent flankers (*P* = 0.005), Dunn *post hoc* test showed that D-prime was larger for adults [mean (SD); 1.67 (0.61)] than for elderly [0.97 (0.47); *P* = 0.004)].

##### D Prime—Multivariate Analyses

**Figure 4:**
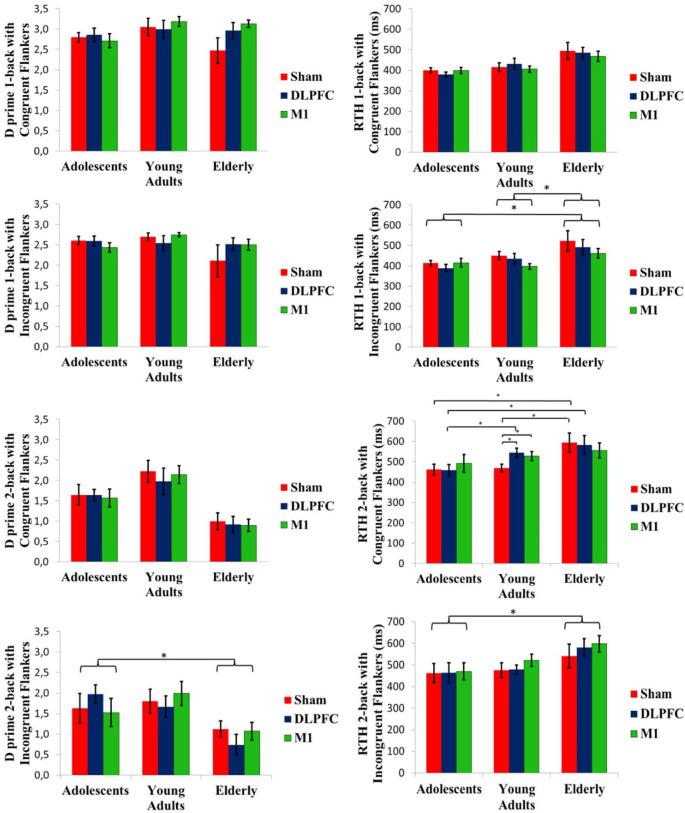
D prime and RTH at the n-back task conducted after the stimulation. Asterisks indicate statistical significance (*P* < 0.05). Error bars represent SEM. Brackets represent the main effect of age group differences.

The GEE model between stimulation condition and age groups showed no stimulation effect on the D-prime measure, within or between age groups. Baseline D-prime was included as a covariate on GEE models. There was a significant group main effect for D-prime at the two-back task with incongruent flankers (Wald *χ*^2^ = 12.572; *df* = 2; *P* < 0.01). Adolescents showed a higher D-prime in this condition than the elderly (*P* < 0.01; Figure 4). However, the interaction of group and stimulation was not significant. An order effect was not observed for one-back with congruent flankers (Wald *χ*^2^ = 5.041; *df* = 2; *P* = 0.08), neither for one-back with incongruent flankers (*Wald*_(2)_ = 5653, *P* = 0.05) and nor for two-back with incongruent flankers (Wald *χ*^2^ = 3.039, *df* = 2; *P* = 0.21). For two-back with congruent flankers, there was a significant order effect (Wald *χ*^2^ = 9.786, *df* = 2; *P* < 0.01), *post hoc* test showed that the first cross-over session had a lower D-prime than the third session (*P* < 0.01).

##### RTH—Univariate Analyses

Mean baseline RTH was compared by the Kruskal-Wallis test. It revealed a significant difference for one-back with congruent flankers (*P* = 0.02). Dunn post-hoc test showed that adolescents [mean (SD), 391 ms (50)] performed faster than the elderly [488 ms (87), *P* = 0.02].

##### RTH—Multivariate Analyses

A GEE model showed a significant interaction of age group and stimulation condition for the two-back task with congruent flankers (Wald *χ*^2^ = 16.068, *df* = 4; *P* < 0.01). Post-hoc test showed that for the sham tDCS, adolescents and adults had a shorter RTH than the elderly (*P* < 0.01 for both). While for a-tDCS over DLPFC, adolescents had a faster RTH compared to either adults or elderly (*P* < 0.01 for both), whereas stimulation over M1 produced a faster RTH for adolescents when compared to elderly (*P* = 0.03). Within the adults’ group, the sham condition had a faster RTH compared to a-tDCS, either on the DLPFC or M1 (*P* < 00.1 for both; Figure 4). For the one-back task with incongruent flankers, a main effect of group was significant (Wald *χ*^2^ = 9.147, *df* = 2; *P* = 0.01). Adults and adolescents showed a faster RTH than the elderly (P *=* < 0.01 and *P* = 0.03, respectively). The RTH for the two-back task with incongruent flankers also showed the main effect of the group (Wald *χ*^2^ = 11.822, *df* = 2; *P* < 0.01). Adolescents had shorter RTH than the elderly (*P* < 0.01; Figure 4). Order effect was not observed for RTH for one-back with congruent flankers (Wald *χ*^2^ = 1.34; *df* = 2; *P* = 0.51), neither for two-back with incongruent flankers (Wald *χ*^2^ = 4.89; *df* = 2; *P* = 0.08), but significant for two-back with congruent flankers (Wald *χ*^2^ = 12.371; *df* = 2; *P* < 0.01) with a faster RTH for the third session compared to the first (*P* < 0.01), and for one-back with incongruent flankers (Wald *χ*^2^ = 6.29;*df* = 2; *P* = 0.04) *post hoc* not significant.

## Discussion

These findings revealed that the tDCS effect on pain perception and working memory depends on the stimulation site and that such effect is mediated by age. The a-tDCS over the DLPFC reduced the HPT in adolescents, in contrast, no significant difference was found for HPT in adults and elderly for DLPFC tDCS. Likewise, for adults, a-tDCS over M1 increased HPT. However, in the elderly, we did not find a significant difference in the HPT despite the stimulation site. Concerning RTH on the n-back task, analysis within-group in adults showed an increased reaction time for the two-back task with congruent flankers in the a-tDCS compared to sham in both stimulation sites (DLPFC and M1). In summary, these results strengthen the importance of considering age as a mediator that can change the tDCS effect according to the stimulation area and it likely is involved in the inter-subject response variability. Their importance is to lay the initial steps to investigate and to consider age as an appropriate stimulation parameter to use the tDCS.

A-tDCS on the DLPFC in adolescents displayed an increase in pain perception. Although there is not a clear explanation for this increase, it suggests that age influences the inter-group variability of the tDCS response. From a physiological point of view, this convergent effect of tDCS on pain measures (e.g., HPT, HPTol, HMP) supports the hypothesis that there is a distinct impact of the a-tDCS on the DLPFC in adolescents compared to two other age groups. It is plausible that the system can have suffered an overload of excitatory stimulus when combined with the a-tDCS effect across 20 min with an online WM task at last 10 min of tDCS session, and thus, it drove to a protective inhibitory response. This is plausible since the sham effect in the same group did not provide such a response. These findings support the hypothesis that this distinct effect related to the DLPFC can be linked to an increase in either the excitability or the state-dependent neuroplasticity. This hypothesis can find some support from the results observed in the two other age groups, which showed the a-tDCS effect tends to decrease pain perception on the DLPFC. This tendency in the direction to improve pain perception is also in agreement with a result found in adult women with fibromyalgia with an extended home-based a-tDCS use, which demonstrated an improvement in the pain and the disability due to pain (Brietzke et al., 2019). Thus, the main result of this research extends literature that age is a mediator of the neuroplasticity state involved in the a-tDCS on the DLPFC in adolescents. It is important to realize that these results contrast with our initial hypothesis that the top down modulation with a-tDCS could improve the pain perception. We assume that this reverse effect could be a protective physiological response. Aligned with this perspective are trends found on the a-tDCS effect on the M1 on pain measures in adolescents. It tends to reduce pain perception and indicates an effect likewise that was found in adults. In summary, these results permit us understanding the role of age as mediators of tDCS. However, they do not support therapeutic decision-making in clinical settings.

These results could be a proof of concept that age is a central mediator of the tDCS response, particularly for DLPFC, and that age can be a determinant of non-linear state-dependence response and explains at least part of individual differences of the tDCS effect, despite identical stimulation parameters (Hsu et al., 2016). This hypothesis finds support in neurophysiological measures that showed that intracortical inhibition increases with age (Croarkin et al., 2014). This way, it is possible to hypothesize that the hyper neuronal plasticity in the circuitries at baseline did not support the additional load of the a-tDCS excitatory stimulus applied over the DLPFC. This way, the decrease in the pain threshold could be interpreted as a physiological contra-regulatory response to protect the system of dysfunctions by excessive excitability and it can be a physiological protective reaction in front of a demand that crosses the limit to effort at an adaptation of this neuronal circuitries. This assumption is substantiated by the previous study using cathodal tDCS protocol, which enhanced cortical excitability in adolescents (Moliadze et al., 2015). Additional factors involved in this response are a larger electrical field for children and adolescents due to parameters like smaller skull thickness and increased cerebrospinal fluid volume when compared to adults (Minhas et al., 2013). According to an earlier study, intensified a-tDCS protocols can shift directionally of effects (Monte-Silva et al., 2013). This way, the increase in the pain sensitivity indexed by the HPT in adolescents when the stimulation was over the left DLPFC might have caused by an inhibitory tDCS effect. Another possible explanation for this result could be related to the left DLPFC stimulation that enhanced attention to the painful stimulus resulting in higher pain perception. This hypothesis finds some support in a previous study in adolescents with attention deficit hyperactivity disorder, where an improvement in the attention with a-tDCS over the left DLPFC was observed (Soff et al., 2017).

The increase in the pain threshold in adults with tDCS applied over M1 is supported by studies using similar protocols (Boggio et al., 2008; Reidler et al., 2012; Zandieh et al., 2013). However, our findings related to the HPT in adults with the stimulation over the left DLPFC diverge from a previous study, which found a significant increase in HPT after DLPFC stimulation (Boggio et al., 2008). Possible explanations for these conflicting results are sample characteristics and methodological aspects: in the mentioned study, the adults’ group is younger than the group of the same category in the current study. There are additional differences related to protocol, such as the time of stimulation, which they applied for 5 min as well as the pain threshold evaluation performed online after 3 min of stimulation. Another study found that in healthy subjects, the a-tDCS over DLPFC increased tolerance to heat pain, but not the pain threshold (Mylius et al., 2012). Likewise, our results in adults with stimulation over M1 are aligned with a meta-analysis on healthy subjects which found increased pain thresholds (Vaseghi et al., 2014). Moreover, a recent meta-analysis evaluated the impact of a-tDCS on diverse chronic pain conditions and found more consistent evidence when the stimulation was applied over M1, even though it suggests that it can be effective for pain treatment when applied over DLPFC (Zortea et al., 2019).

Thus, the biological plausibility to support our result might be related to different connections of M1 and DLPFC within areas of pain processing. The M1 is associated with lateral thalamic nuclei that are involved in the sensory discrimination aspects of pain, whereas the DLPFC is associated with medial thalamic nuclei and the limbic system, which are associated with emotional and cognitive aspects of pain (Boggio et al., 2008). Thereby, perhaps it improved the function in neural networks responsible for the cognitive and emotional components of pain. These distinct effects observed, according to the area of stimulation, are supported by neuroimage studies (Morton et al., 2016). Such studies showed the tDCS effect could modify cortical and subcortical networks activated by pain. Among them are the primary somatosensory cortex (S1), and the secondary somatosensory cortex (S2). These areas encode sensory features of pain, such as the location and duration of pain (Apkarian et al., 2005). Alternatively, however, it also involves the ACC and insula, which are associated with emotional aspects of pain (Apkarian et al., 2005; Dum et al., 2009). Also, the plausibility of the effect observed in DLPFC stimulation is supported by anatomical and neurophysiological features, since the prefrontal cortex is a critical structure for attention and executive functions (Fuster, 2008). The prefrontal cortex also modulates the inhibition of neuronal coupling along the ascending midbrain-thalamic-cingulate pathway (Lorenz et al., 2003).

Additionally, we did not find a significant effect of any a-tDCS protocol on HPT within the elderly group. The difference in HPT between the elderly and adults can be related to age-dependent neuroplasticity decline in brain areas associated with pain processing. Given in the elderly compared to young adults display significantly lower gray matter perfusion, a lower gray matter density as well as lower brain metabolism as measured by oxygen consumption (Sowell et al., 2004). Animal studies at the cellular level showed that the synaptic plasticity is impaired with the senescence, especially concerning the decline of LTP (Barnes, 1979; Rex et al., 2005). Furthermore, there is the age-dependent cerebral brain atrophy, which increases scalp-to-brain distance (Resnick et al., 2003), which might also reduce the tDCS effect. Moreover, we cannot rule out, at least partially, that the result in the elderly could be mitigated by the assessment of HPT immediately after the stimulation end. This assumption finds some support in the literature because the tDCS effect in the elderly can be delayed for up to 30 min to induce the neuroplasticity process (Fujiyama et al., 2014). We can state that these findings help to comprehend the physiological process, and they should not be generalized to clinical protocols with multiple sessions. This argument is supported by earlier studies in the elderly with chronic pain when identical stimulation parameters on the M1 during 5 or 10 sessions were effective to improve pain scores (Harvey et al., 2017; da Graca-Tarragó et al., 2019).

Our results concerning the tDCS effect on WM accuracy agree with those of previous studies. Recent meta-analyses discuss that the impact of a-tDCS over the DLPFC in healthy adults appears to be restricted to reaction time (Brunoni and Vanderhasselt, 2014; Dedoncker et al., 2016; Hill et al., 2016). In contrast, another meta-analysis found a small, but significant effect, of anodal tDCS over the left DLPFC on performance accuracy when tDCS was combined with online WM training (Mancuso et al., 2016), or when it was applied in multiple stimulation sessions (Mashal and Metzuyanim-Gorelick, 2019). Moreover, earlier studies observed the effect of tDCS only when the task included a three-back (Fregni et al., 2005; Ohn et al., 2008; Gill et al., 2015), a more difficult task compared to the two-back conducted in the present study. Therefore, a potential explanation for the lack of stimulation effect in the WM performance was a ceiling effect.

The most common adverse effects reported were itching and sleepiness with a similar incidence in active and sham stimulation conditions. However, for the DLPFC protocol, we did find a significant difference in the intensity of itching: adolescents reported moderate intensity, while adults and elderly majorly reported mild effects. Serious adverse effects were not reported. The adverse effects observed in our study, like those that have been published, when present, were transient and most classified as mild.

### Study Limitations

Some limitations related to the study design need to be considered. First, although we need parsimony in regards to the generalizability since it is an explanatory proof-of-concept trial and that the impact of the intervention was evaluated in healthy women using tightly controlled methods where there is an attempt to maximize the internal validity and assay sensitivity (i.e., effect detection ability; Gewandter et al., 2014). Differences between sex on pain processing revealed that women, compared to men, showed a higher medial prefrontal activation under nociceptive stimuli (Gupta et al., 2017). Also, in women, we found a higher inhibitory function of the descending pain modulating system compared to males (Gasparin et al., 2020). Additionally, there is vast literature related to sex differences in pain sensitivity (Bartley and Fillingim, 2013). Also, studies have indicated that there is an influence of sex on the tDCS effect (Kuo et al., 2006; Ohn et al., 2008). Second, the phase of the menstrual cycle could influence pain processing and cortical excitability, however, the use of hormonal contraceptive methods can suppress hormonal cycle fluctuation. Third, elderly subjects were not screened for mild cognitive impairment. Approximately 4.5% of the elderly have mild cognitive impairment (Sachdev et al., 2015), which is associated with deficits in WM performance compared to age-matched controls (Kirova et al., 2015). Nevertheless, we did not observe differences in the tDCS on WM using a similar protocol in all three age groups. This result is aligned with a study showing that n-back accuracy did not elicit a result with a significant difference between healthy controls and the elderly with minor cognitive impairment (Kochan et al., 2010; Emonson et al., 2019). Fourth, we found a difference between age groups in the educational level. Thereby, all analyses of the tDCS effect on the working memory were adjusted by the educational level. Thus, it is improbable that this difference changes the directions of our conclusions. Fifth, the order of sessions was significant for a few WM outcomes. A 7-day wash-out is adequate to prevent carry-over effects (Nitsche et al., 2008). The difference in the order can be related to the learning effect. However, the order of the sessions was added as a covariate for the GEE model to consider possible order effects. Additionally, crossover design can help avoid the overestimation of the benefits of the intervention being tested (Mills et al., 2009). Sixth, evaluators were not blinded; although lack of blinding is associated with performance bias, the outcomes of QST and n-back by the test characteristics are less prone susceptible for evaluators’ influence than self-report measures (Higgins et al., 2011). Finally, this is an exploratory study with small sample size, thus there is an increased chance for type I and type II errors, and thus the results require replication with larger sample sizes to be able to make more firm conclusions. Although this study has such limitations, from a clinical standpoint, these results give additional data to plan further studies to obtain evidence about the impact of age as a mediator factor on the tDCS effects according to the electrode positions in confirmatory trials in clinical conditions (i.e., pain, cognitive rehabilitation, etc.).

## Conclusion

In conclusion, these findings suggest that a-tDCS modulates pain perception and WM differentially according to age and target area of stimulation. In adolescents, anodal stimulation on the DLPFC increased the pain perception, while in adults, the stimulation on the M1 increased the pain threshold. Thus, they elucidate the impact of tDCS for different age groups and can help to define according to age what is the appropriate intervention in further clinical trials.

## Data Availability Statement

The raw data supporting the conclusions of this article will be made available by the authors, without undue reservation.

## Ethics Statement

The study was reviewed and approved by Institutional Review Board of the Hospital de Clínicas of Porto Alegre (IRB HCPA/Approval number: 170188). Written informed consent to participate in this study was provided by the participants’ legal guardian/next of kin.

## Author Contributions

JS, MZ, and WC conceived and designed the study, participated in the data collection, performed the statistical analysis, and coordinated and drafted the manuscript. CD participated in the data collection and registering. FF helped conceive and design the study. IT, M-FK, and MN reviewed the manuscript.

## Conflict of Interest

MN is in the scientific advisory boards of Neuroelectrics, and NeuroDevice. The remaining authors declare that the research was conducted in the absence of any commercial or financial relationships that could be construed as a potential conflict of interest.

## Footnotes

^1^https://www.ncss.com
