# Supplementary material for: Impact of Age on tDCS Effects on Pain Threshold and Working Memory: Results of a Proof of Concept Cross-Over Randomized Controlled Study
Source: Front Aging Neurosci. 2020 Jun 30;12:189. doi: 10.3389/fnagi.2020.00189 (PMC7344165; doi:10.3389/fnagi.2020.00189)
Supplement: Supplementary file 1 [file Data_Sheet_1.PDF]

Supplementary Material

SUPPLEMENTARY TABLES AND FIGURES

Guessing	Adolescents Sham	Adolescents Active-tDCS	Adults Sham	Adults Active-tDCS	Elderly Sham	Elderly Active-tDCS	*P-value*
Intervention							
Sham	2	7	1	9	3	6	
DLPFC	2	7	1	9	1	8	0.52
M1	5	4	0	10	2	7	0.35

Data were analyzed with Fisher`s exact test comparing sham and each active stimulation not individualizing for age group

Table 1. Blinding data shown by guesses of participants if they received sham or active stimulation for all stimulation conditions. There was no statistically significant difference between sham or active conditions for each protocol applied in the study.

**Adverse effects (number of episodes)**	Adolescents Sham n=10	Adolescents DLPFC n=9	Adolescents M1 n=9	***P-value***	Adults Sham n=10	Adults DLPFC n=10	Adults M1 n=10	***P-value***	Elderly Sham n=10	Elderly DLPFC n=9	Elderly M1 n=9	***P-value***
Headache	3	0	1	0.673	0	2	1	0.66	1	1	1	1
Neck pain	2	0	1	1	2	4	4	0.39	0	1	1	1
Scalp pain	2	3	1	0.44	2	2	2	1	1	1	2	0.66
Tingling	6	7	6	0.55	5	3	3	0.57	4	2	6	0.12
Itching	8	8	7	0.52	8	8	8	0.23	4	4	3	0.85
Burning	4	5	5	0.50	4	3	4	0.89	2	2	1	1
Skin redness	2	2	2	1	5	6	6	0.39	1	4	4	0.21
Sleppness	7	4	7	1	9	9	6	0.67	5	4	7	0.66
Trouble concentrating	5	4	5	1	9	6	9	0.53	4	7	5	0.45
Mood acute change	0	1	0	1	0	1	0	1	0	1	1	1

P-value of Fisher´s exact test conducted within age groups for differences of frequencies of adverse effects between tDCS protocols, except headache, which was analyzed via chi square tests.

Table 2. Adverse effects. Data show the number of participants in each age group that reported respective adverse effects. There was no significant difference of the incidence of side effects between active and sham tDCS protocols for all age groups.

Primary outcomes							
	**Adolescents** Mean (SD)	**Adults** Mean (SD)	**Elderly** Mean (SD)	*Age group*	*Order of stimulation*	*Stimulation condition*	*Stimulation area * age group:*
**D-prime one-back with congruent flankers †**							
Sham	2.80(0.3)	3.05(0.6)	2.47(0.9)	*Wald χ^2=2.642*	*Wald χ^2= 5.041*	*Wald χ^2 =2.819*	*Wald χ^2=7.984*
DLPFC	2.85(0.4)	2.99(0.6)	2.96(0.5)	*df=2*	*df=2*	*df=2*	*df=4*
M1	2.71(0.4)	3.18(0.3)	3.13(0.2)	*P=0.260*	*P=0.08*	*P=0.240*	*P=0.095*
Baseline one-back with congruent flankers D-prime : Wald χ^2=7.264; df=1;P=0.007.							
Years of study: Wald χ^2=0.103; df=1;P=0.74							
**D-prime one-back with incongruent flankers**							
Sham	2.60(0.3)	2.70(0.2)	2.11(1.2)	*Wald χ^2=0.439*	*Wald χ^2= 5.653*	*Wald χ^2 =0.246*	*Wald χ^2=5.200*
DLPFC	2.59(0.3)	2.54(0.5)	2.51(0.4)	*df=2*	*df=2*	*df=2*	*df=4*
M1	2.43(0.3)	2.74(0.1)	2.50(0.4)	*P=0.803*	*P=0.056*	*P=0.887*	*P=0.263*
Baseline one-back with incongruent flankers D-prime : Wald χ^2=17.126; df=1;P<0.01.							
Years of study: Wald χ^2=1.019; df=1;P=0.312							
**D-prime two-back with congruent flankers**							
Sham	1.64(0.8)	2.22(0.8)	0.99(0.6)	*Wald χ^2 =8.342*	*Wald χ^2= 9.786*	*Wald χ^2=1.31 4*	*Wald χ^2=1.162*
DLPFC	1.63(0.4)	1.97(1.0)	0.91(0.6)	*df=2*	*df=2*	*df=2*	*df=4*
M1	1.56(0.6)	2.14(0.6)	0.89(0.4)	*P=0.012*	*P=0.000*	*P=0.514*	*P=0.887*
Baseline two-back with congruent flankers D-prime : Wald χ^2=12.365; df=1;P=0.000.							
Years of study: Wald χ^2=1.312; df=1;P=0.255							
**D-prime two-back with incongruent flankers**							
Sham	1.62(1.1)	1.80(0.9)	1.12(0.6)	*Wald χ^2 =12.576*	*Wald χ^2= 3.039*	*Wald χ^2=0.11 6*	*Wald χ^2=3.585*
DLPFC	1.97(0.6)	1.66(0.8)	0.73(0.7)	*df=2*	*df=2*	*df=2.*	*df=4*
M1	1.52(0.9)	1.99(0.8)	1.07(0.6)	*P=0.000*	*P=0.211*	*P=0.942*	*P=0.469*
Baseline two-back with incongruent flankers D-prime : Wald χ^2=18.105; df=1;P=0.001.							
Years of study: Wald χ^2=0.071; df=1;P=0.795							

†GEE model; D Prime (D prime = hit rate minus false alarm rate).

Table 3. N-back derived D-prime according to age groups and stimulation condition. Data are presented as mean and standard deviation (SD) (n=30).

Secondary outcomes							
	**Adolescents** Mean (SD)	**Adults** Mean (SD)	**Elderly** Mean (SD)	*Age group*	*Order of stimulation*	*Stimulation condition*	*Stimulation area * age group:*
**RTH one-back with congruent flankers †**							
Sham	399(036)	415(067)	494(129)	*Wald χ^2=5.710*	*Wald χ^2= 1.342*	*Wald χ^2 =1.231*	*Wald χ^2=7.203*
DLPFC	379(036)	431(083)	485(080)	*df=2*	*df=2*	*df=2*	*df=4*
M1	398(045)	405(047)	468(077)	*P=0.058*	*P=0.511*	*P=0. 540*	*P=0.126*
Baseline one-back with congruent flankers RTH : Wald χ^2=1.658; df=1;P=0.198.							
Years of study: Wald χ^2=4.339; df=1;P=0.370							
**RTH one-back with incongruent flankers**							
Sham	413(040)	449(067)	522(156)	*Wald χ^2=9.147*	*Wald χ^2= 6.295*	*Wald χ^2 =5.496*	*Wald χ^2=8.035*
DLPFC	388(054)	434(081)	491(114)	*df=2*	*df=2*	*df=2*	*df=4*
M1	415(060)	397(041)	460(069)	*P=0.010*	*P=0.043*	*P=0.064*	*P=0.090*
Baseline one-back with incongruent flankers RTH : Wald χ^2=0.077; df=1;P=0.782.							
Years of study: Wald χ^2=8.580; df=1;P=0.003							
**RTH two-back with congruent flankers**							
Sham	461(083)	469(058)	594(150)	*Wald χ^2 =44.366*	*Wald χ^2= 12.371*	*Wald χ^2=1.047*	*Wald χ^2=16.068*
DLPFC	458(085)	544(069)	581(140)	*df=2*	*df=2*	*df=2*	*df=4*
M1	492(123)	528(066)	555(110)	*P=0.000*	*P=0.002*	*P=0.592*	*P=0.003*
Baseline two-back with congruent flankers RTH : Wald χ^2=141.345; df=1;P=0.000.							
Years of study: Wald χ^2=0.603; df=1;P=0.438							
**RTH two-back with incongruent flankers**							
Sham	462(138)	475(108)	541(171)	*Wald χ^2 =11.822*	*Wald χ^2= 4.895*	*Wald χ^2=4.770*	*Wald χ^2=4.023*
DLPFC	463(140)	479(067)	581(124)	*df=2*	*df=2*	*df=2.*	*df=4*
M1	470(112)	522(084)	598(112)	*P=0.003*	*P=0.087*	*P=0.092*	*P=0.403*
Baseline two-back with incongruent flankers RTH : Wald χ^2=13.552; df=1;P=0.000							
Years of study: Wald χ^2=3.535; df=1;P=0.060							

†GEE model; RTH - Response time for hits is presented in milliseconds.

Table 4. N-back derived RTH according to age groups and stimulation condition. Data are presented as mean and standard deviation (SD) (n=30)
